# DriverSubNet: A Novel Algorithm for Identifying Cancer Driver Genes by Subnetwork Enrichment Analysis

**DOI:** 10.3389/fgene.2020.607798

**Published:** 2021-02-19

**Authors:** Di Zhang, Yannan Bin

**Affiliations:** ^1^College of Information Engineering, Shaoguan University, Shaoguan, China; ^2^Institutes of Physical Science and Information Technology, Anhui University, Hefei, China

**Keywords:** cancer, driver gene, multi-omics data, neighbor network, TCGA

## Abstract

Identification of driver genes from mass non-functional passenger genes in cancers is still a critical challenge. Here, an effective and no parameter algorithm, named DriverSubNet, is presented for detecting driver genes by effectively mining the mutation and gene expression information based on subnetwork enrichment analysis. Compared with the existing classic methods, DriverSubNet can rank driver genes and filter out passenger genes more efficiently in terms of precision, recall, and F1 score, as indicated by the analysis of four cancer datasets. The method recovered about 50% more known cancer driver genes in the top 100 detected genes than those found in other algorithms. Intriguingly, DriverSubNet was able to find these unknown cancer driver genes which could act as potential therapeutic targets and useful prognostic biomarkers for cancer patients. Therefore, DriverSubNet may act as a useful tool for the identification of driver genes by subnetwork enrichment analysis.

## Introduction

Cancer is a globally prevalent threat to the overall survival of patients, and is driven by a few important cancer genes, viz., driver genes (Dinstag and Shamir, 2019). Oncogenic mutations on driver genes contribute to abnormal cell proliferation and tumor development. Most other genes undergoing mutations due to genomic instability caused by driver genes, termed passenger genes, are neutral, and do not lead to any cancerous growth (Di Zhang et al., 2016; Yue et al., 2018). Thus, increasing efforts are being made to recognize these driver genes for developing a better elucidation regarding cancer initiation and progression. There are some systemic cancer genomics research projects, such as The Cancer Genome Atlas (TCGA), which is a public free platform and provides data on 33 cancer types for cancer research.

Computational patterns have been developed to screen driver genes by distinguishing them from passenger genes through mutation frequency. For instance, MuSiC adopts a statistical approach to detect driver genes with significantly high mutative rates (Dees et al., 2012). DeepDriver employs deep learning to identify driver genes by estimating the functional impact of mutations (Luo et al., 2019). However, these methods are based on mutation frequency, and do not uncover driver genes which carry few variants. Recently, researchers realize that genes cooperate with each other in cancer progression through biological pathways, and detection of driver genes by pathway- or network-based pipelines is emerging with a high speed (Hou et al., 2018). These studies revealed that functional networks could be available for identifying driver genes without consideration of mutation frequency. They concentrate on uncovering cancer associated core modules consisting of gene-sets rather than a single gene critical to tumor progression. The lack of prioritization in this approach is a shortcoming from the considerations of clinical treatment, particularly when the predicted driver gene set contains more than one gene.

To solve this situation, many algorithms have been developed to rank the candidate genes (Hou and Ma, 2014; Dinstag and Shamir, 2019; Hristov et al., 2020). For instance, HotNet2 identifies rare mutations across pathways and protein-protein interaction (PPI) networks using the heat-diffusion theory (Leiserson et al., 2015). DriverNet also consolidates PPI and gene expression data to uncover driver genes (Bashashati et al., 2012). DawnRank method adopts Google's PageRank algorithm and ranks an individual's mutated gene profile by means of measuring the effect of each mutated gene on the differentially expressed genes (DEGs) (Hou and Ma, 2014). MUFFINN algorithm evaluates the significance of mutations on neighboring genes in the specific network, demonstrating excellent predictive performance in a large number of patients (Cho et al., 2016). MaxMIF tries to find driver genes by evaluating the impact of single nucleotide variants on transcriptional networks (Hou et al., 2018). Nevertheless, the false positive rates of the current existing computational algorithms need to be further reduced.

Here, we have designed an effective algorithm, called DriverSubNet, which has the ability of prioritizing driver genes. In this approach, the driver genes were scored by combining their influence on DEGs in each neighbor subnetwork and their mutation frequency. These pipelines are based on enrichment of subnetworks, where each subnetwork may reflect the situation of dysregulated biological process in tumor. Thus, the extent to which a given driver gene explains multiple functional biological process deregulations serves as a proxy for the likelihood that this gene is indeed the driver. Our algorithm views that driver genes affect the deregulations of other genes in the functional biological processes. Besides, mutation recurrence makes a vital contribution on detecting high frequency mutated drivers. In fact, the true cancer drivers have good connectivity to these functional biological processes, and our algorithm aims to measure such connections directly via subnetwork enrichment and the impact of mutations.

## Materials and Methods

### Data Collection

For four cancer types, including thyroid carcinoma (THCA), kidney renal clear cell carcinoma (KIRC), and breast cancer (BRCA) and Head-Neck Squamous Carcinoma (HNSC), somatic mutations, somatic copy number alterations (SCNAs), and RNA-seq expression data belong to the TCGA (Weinstein et al., 2013) platform, downloaded from the UCSC data portal (http://xena.ucsc.edu/) (Rosenbloom et al., 2015). Undirected interaction network information was collected from the Human Protein Reference Database (HPRD) release 9 (Keshava Prasad et al., 2009). HPRD is a comprehensive resource for studying the human proteome, and the proteins have been manually extracted from the literature by expert biologists. In the mutation matrix, where a row denotes a gene, and a column denotes a patient, if a gene exists the mutations (e.g., SCNAs, small insertions, and small deletions), which was marked as one, otherwise marked as zero. Gene expression profiles from control samples were also used for differential expression analysis. The details of the data can be seen in Supplementary Table 1. To evaluate the performance of our results, we obtained the set of all 723 entries from the Cancer Gene Census (CGC, Accessed on: 01/30/2020) (Tate et al., 2019). Functional gene sets were collected from literature (Ge et al., 2018; Malta et al., 2018; Sanchezvega et al., 2018) and the Atlas of Cancer Signaling Network website (https://acsn.curie.fr/ACSN2/ACSN2.html), which includes data for various pathways including ubiquitin pathway, DNA repair pathway, TGF-beta signaling, and oncogenic signaling pathway. Finally, we used the Functional Set (FG) with 3,681 functional genes to represent the functional biological processes.

### Evaluation Criteria

The performance of algorithms for prioritizing candidate genes was widely adopted the following criteria: precision, recall, and the F1 score (Bashashati et al., 2012; Hou and Ma, 2014). MUFFINN, Dawnrank, and DriverNet were the state of art methods to be compared with other algorithms. We use MUFFINN algorithm based on NDmax and HumanNet. One hundred top-ranked candidate genes were selected to compare the state-of-art methods (Hui et al., 2019). The following evaluation criteria were used to assess the ability of a method to identify real driver genes from the top-ranked candidates.

$$\begin{array}{l} Precision=\frac{\left(\#\;Genes\;in\;CGC\right)\cap \;\left(\#Genes\;found\;in\;our\;method\right)}{\left(\#Genes\;found\;in\;our\;method\right)}\\ \;Recall=\frac{\left(\#Genes\;in\;CGC\right)\cap \left(\#Genes\;found\;in\;our\;method\right)}{\left(\#Genes\;in\;CGC\right)}\\ \;F1\;sore=2\times \frac{Precision\times Recall}{Precision+Recall} \end{array}$$

### Scoring Scheme of DriverSubNet

**Figure 1:**
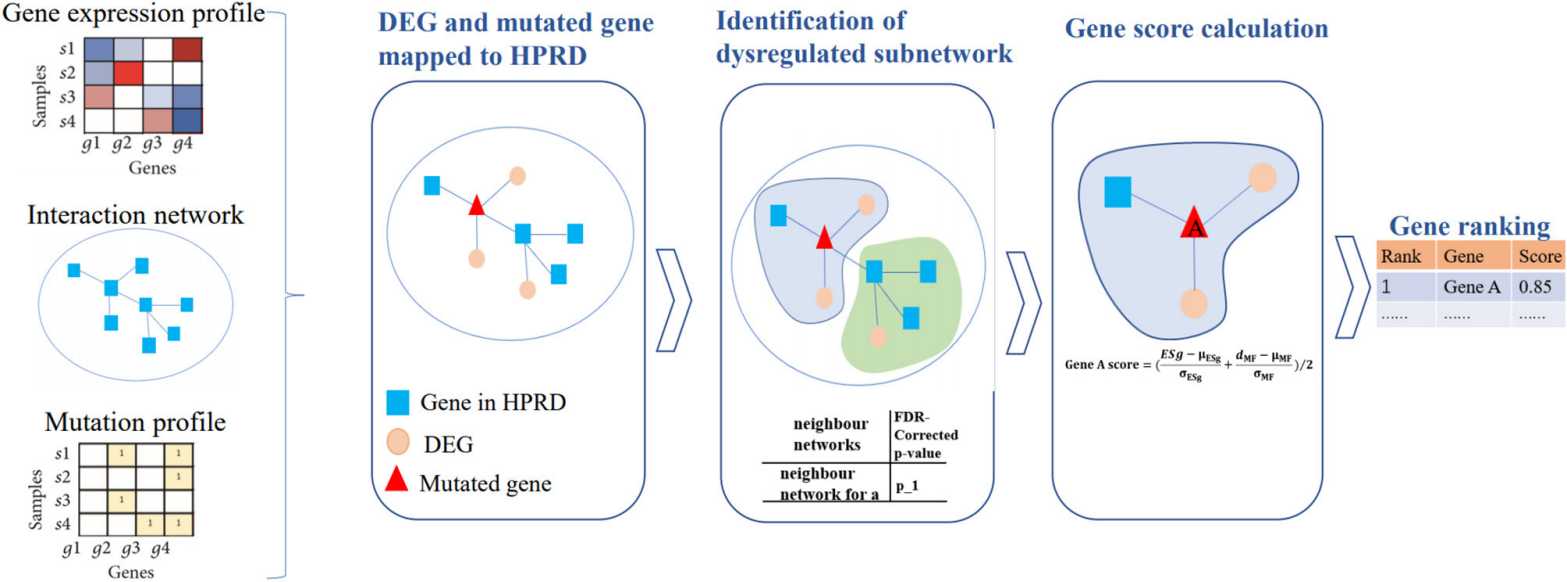
The pipeline of DriverSubNet. Differential expression analysis was performed using the DESeq2 package. All genes with adjusted *p* < 0.05 were considered as DEGs. DEGs and mutated genes were mapped to the network. We extract a neighbor subnetwork from the protein interaction network for each mutated gene, where a gene and its neighbor genes together consisted of an adjacent neighbor subnetwork. We then evaluate whether the subnetwork is deregulated subnetwork by enrichment analysis. An enrichment score ESg was obtained by evaluating the functional significance of the central gene in each neighbor subnetwork. Each center gene with an enrichment score *ESg* was calculated. Finally, candidate genes were ranked according to their overall mutational influence scores and *ESg*.

A schematic diagram of our DriverSubNet pipeline consists of four steps (Figure 1). Firstly, differential expression analysis was carried out statistical analysis by using the DEseq2 package in R (version 3.6). All genes with adjusted *p* < 0.05 were considered as DEGs. Secondly, DEGs and mutated genes were mapped to HPRD interaction network. For each mutated gene in HPRD network, mutated gene and its directly connected neighbor genes consist of the adjacent neighbor subnetwork, and the central gene is mutated gene in subnetwork. Thirdly, for each subnetwork, we want to evaluate whether the subnetwork have an impact on vital biological process. For DEGs in subnetwork, we measure whether these DEGs were enriched the FG. If these DEGs were significantly enriched FG, it represents that the subnetwork tends to play a crucial role in cancer progression. In our result, enrichment *p*-value of DEGs was set as 5E-6 across four datasets and the recall value of recognizing known cancer genes in the top 100 genes achieved high. If the enrichment *p*-value of DEGs <5E-6 and the subnetwork consist of more than two genes, the subnetwork was regarded as a deregulated subnetwork. To assess the impact of mutated gene in the deregulated subnetwork, we calculated the mutated impact score *ESg*. We performed the enrichment analysis using the fisher.test function in R (version 3.6), and then transformed it using -log function. It was computed as follows:

$$\begin{array}{l} ESg=-log\left(1-\overset{m-1}{\underset{i=0}{\sum }}\frac{\left(\begin{array}{c} M\\ i \end{array}\right)\left(\begin{array}{c} N-M\\ n-i \end{array}\right)}{\left(\begin{array}{c} N\\ n \end{array}\right)}\right) \end{array}$$

where *N* represents the total genes in each subnetwork, *n* represents the number of DEGs in the subnetwork, *M* represents the overlap with DEGs and functional gene set in each subnetwork, and *i* represents the overlap with DEGs and functional gene set.

Then, in view of combing the effect of gene expression and gene mutations can improve the performance of algorithms (Hou and Ma, 2014), and mutation recurrence makes a vital contribution on detecting high frequency mutated drivers, we also considered mutation frequency in our approach to uncover the most functional drivers in a large number of patients. We evaluated the significance of mutated genes based on the mutation frequency. We calculate the number of mutations according to the mutation matrix, then we normalized the number of mutations, then the value is range 0–1. Finally, we computed a score for every candidate gene by averaging the normalized ESg gene score in the deregulated subnetwork and the normalized gene mutational scores. Candidate genes were ranked according to their overall scores. The score of candidate driver gene score was calculated as follows:

$$\begin{array}{l} Score=\left(\frac{ESg-\mu_{ESg}}{\sigma_{ESg}}+\frac{d_{MF}-\mu_{MF}}{\sigma_{MF}}\right)/2 \end{array}$$

where μ_*ESg*_ is the expected mean of *ESg*, and σ_*ESg*_ is the standard deviation of *ESg*, *d*_*MF*_ is the number of patients with mutated genes, μ_*MF*_ is the expected mean of *d*_*MF*_, and σ_*MF*_ is the standard deviation of *d*_*MF*_.

### Functional Enrichment Analysis

To understand the features detected in our results, we used the R package and found significant enrichment of these uncovered top 100 genes in terms of biological process. Briefly, biological process terms were annotated according to statistical significance. Enrichment was calculated through the hypergeometric test with *p* < 0.05, and following which top 100 most significant categories were selected.

### Survival and Drug Analysis

We used the online tool for analyzing patient survival via its standard processing pipeline GEPIA (Zefang et al., 2017). The drug information for genes was obtained from the Drug Gene Interaction database (DGIdb) (Cotto et al., 2018). DGIdb is comprehensive catalog of druggable genes (i.e., genes with directed pharmacotherapy) and drug-gene interactions database, which integrates existing 30 sources (DrugBank, PharmGKB, Chembl, Drug Target Commons, TTD, and others) and collects 56,309 drug-gene interactions. Drug-gene interactions represents that genes or gene products are known or predicted to interact with drugs, and the gene might be targeted therapeutically. In our study, we use DGIdb to analyze whether these identified genes are clinically relevant genes.

## Results

### Performance Evaluation for Known Cancer-Related Genes

Here, we adopt a subnetwork analysis with PPI information. The core of algorithm is a local subnetwork model, which views that a driver gene can be detected by aggregating its involvement in functional biological process from a central gene and its direct neighbor DEGs. We applied DriverSubNet to four datasets from BRCA, THCA, KIRC, and HNSC, respectively, which the cancer type is randomly chose. Then, we evaluate the effectiveness of our method, MUFFINN, Dawnrank, and DriverNet algorithms.

**Figure 2:**
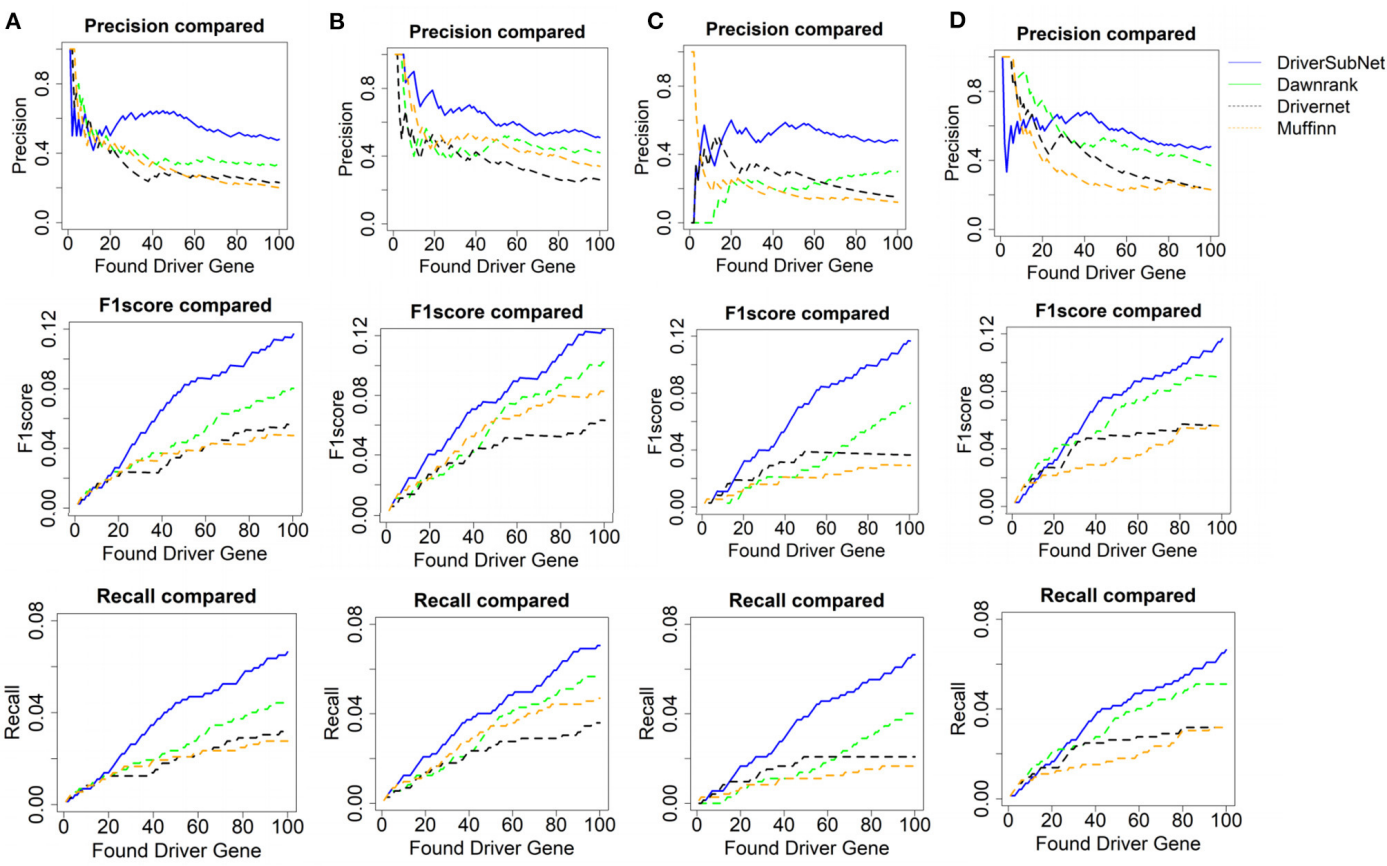
Performance comparison with CGC in terms of precision, *F*1 score, and recall of Dawnrank and DriverNet methods on **(A)** BRCA, **(B)** HNSC, **(C)** KIRC, and **(D)** THCA datasets.

The performances of DriverSubNet, MUFFINN, Dawnrank, and DriverNet methods were compared on the basis of precision, recall, and F1 scores for the top 100 genes. In general, DriverSubNet outperformed MUFFINN, Dawnrank, and DriverNet methods in all four cancer datasets with gold standard CGC dataset (Figure 2). Especially the most of candidate genes were overlapped with CGC in the top 100 driver genes using the DriverSubNet method across four datasets. It suggests that DriverSubNet is robust and has an excellent ability of identifying driver genes. Although the Dawnrank method performed better ability than other algorithms in ranking the top 12 genes in THCA, it had a poorer ability in KIRC. The reason for this phenomenon may be the different number of gene mutations and the variety of gene expression levels across the four cancer types. DriverSubNet is easier to evade the number of mutation noise and expression than other methods. For example, DriverSubNet was able to recover most of known cancer driver genes in the top 100 detected genes across four datasets, while the percentage of known cancer driver genes in the top 100 detected genes using Dawnrank and DriverNet is sensitive to cancer type. This may lead to Dawnrank have a good performance in THCA, while bad performance in KIRC. In KIRC, although some known drivers were found by these three methods, DriverSubNet uncovered significant famous driver genes, such as *EGFR*, which was ranked the 16th, and it were not detected by either Dawnrank or DriverNet or MUFFINN method as the top ranking drivers.

### Novel Candidate Genes Predicted by DriverSubNet

To evaluate the performance of algorithm, precision, recall, and F1 score are widely used to analyze the top 100 genes. In our result, we identified some genes that were not known cancer driver genes. It is essential to explore whether these genes have a potential relationship with cancer. Previous study has suggested that high-ranking unknown cancer driver genes have a potential to be novel driver genes (Hou and Ma, 2014). In our study, we used the top 10 genes to detect some unknown cancer driver genes which have a potential to be novel driver genes.

**Figure 3:**
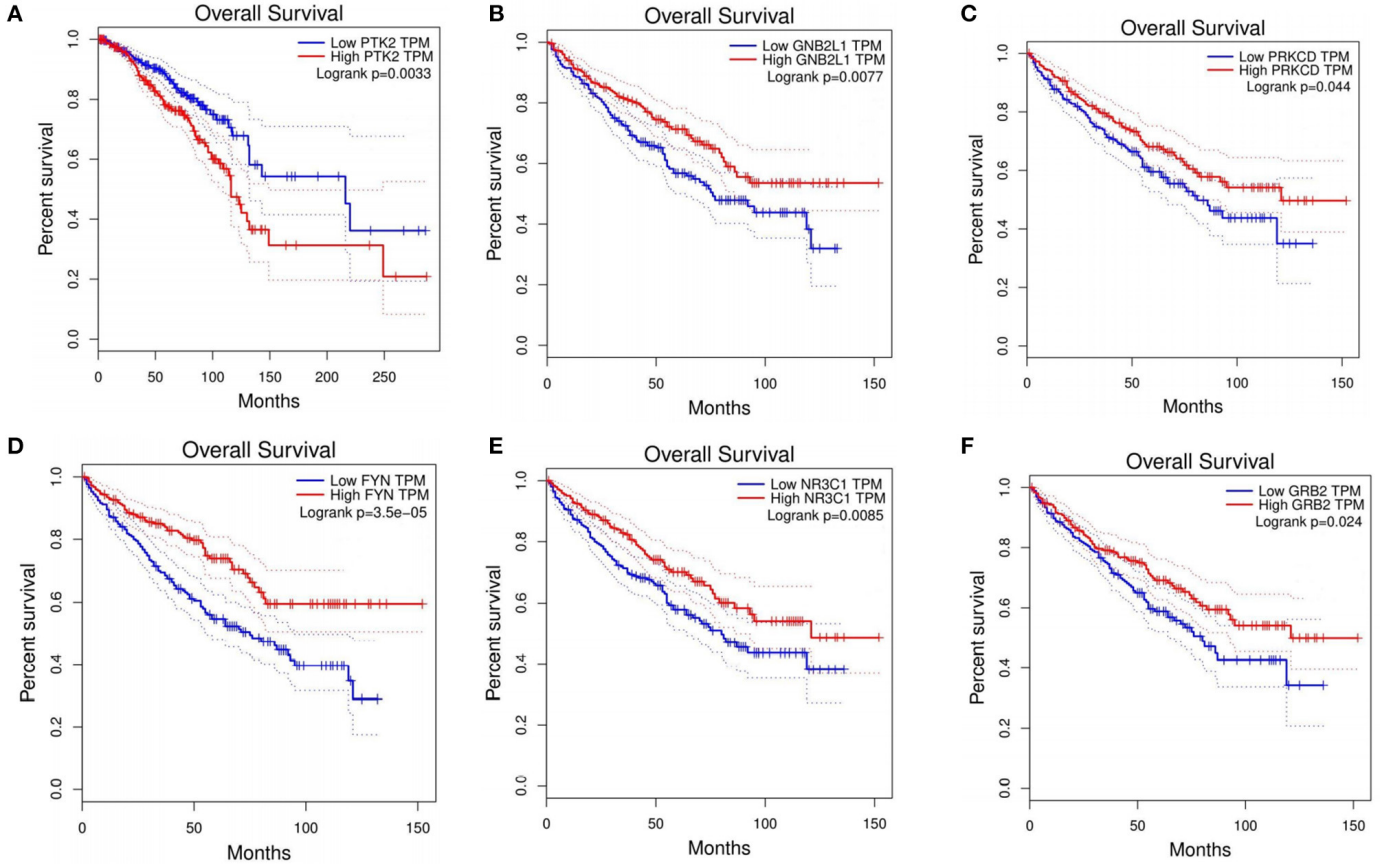
Prognostic value of six genes in cancer patients. **(A)** *PTK2* in BRCA. **(B)** *GNB2L1* in KIRC. **(C)** *PRKCD* in KIRC. **(D)** *FYN* in KIRC. **(E)** *NR3C1* in KIRC. **(F)** *GRB2* in KIRC.

For the BRCA dataset, 48 genes overlapped with CGC for the top 100 candidate driver genes (Supplementary Table 2). Among the top 10 ranking genes in BRCA, *CREBBP, EP300, MYC, SRC*, and *TP53* overlapped with the cancer genes in CGC, whereas the other five genes, (*CDK1, GRB2, YWHAZ*, SHC1, and *PTK2*) did not include in CGC. These five genes were differentially expressed in BRCA. To investigate whether these five genes were involved in BRCA, we explored the correction between these five genes and overall survival in BRCA. Through Kaplan-Meier analysis using an online GEPIA, *PTK2* showed high expression was corrected with a shorter overall survival in BRCA patients (Figure 3A). *CDK1, GRB2*, and *PTK2* were the druggable genes in DGIdb. We concluded that *CDK1, GRB2*, and *PTK2* were more likely to be involved in pathogenesis of BRCA, simultaneously, which have a great potential to be therapeutic targets. Through analysis, *PTK2* can be applied to predict survival of BRCA patients.

For the HNSC dataset, 51 genes overlapped with the genes in CGC for the top 100 candidate driver genes (Supplementary Table 2). Among the top 10 ranking genes in HNSC, *CREBBP, CTNNB1, EGFR, EP300*, MAPK1, *SMAD2, SMAD3, SRC*, and *TP53* overlapped with the genes in CGC, whereas the other one *GRB2* did not. To investigate whether *GRB2* was involved in HNSC, we explored the correction between *GRB2* and overall survival in HNSC. Through Kaplan-Meier analysis, *GRB2* was not corrected with shorter overall survival in HNSC patients. *GRB2* was the druggable gene in DGIdb and more likely to be involved in the pathogenesis of HNSC.

For the KIRC dataset, 48 genes overlapped with CGC for the top 100 candidate driver genes (Supplementary Table 2). Among the top 10 ranking genes in KIRC, *CTNNB1, EP300, SRC*, and *TP53* were found in CGC. Other six genes (*PRKCA, PRKCD, GNB2L1, FYN, NR3C1*, and *GRB2*) did not present in CGC. To investigate whether these genes were involved in KIRC, we explored the correction between these six genes and overall survival in KIRC. Through Kaplan-Meier analysis, five out of the six genes (*PRKCD, GNB2L1, FYN, NR3C1*, and *GRB2*) showed high expression were corrected with shorter overall survival in KIRC patients (Figures 3B–F). It was concluded that these five genes had a great ability to participate in pathogenesis of KIRC, and were possible therapeutic targets. Besides, through the analysis, these five genes can be applied to predict the overall survival of KIRC patients.

For the THCA dataset, 48 genes overlapped with the genes in CGC for the top 100 candidate driver genes. The top 10 ranking genes in THCA were accessed in the Supplementary Table 2. Among these genes, *BRAF, CREBBP, EGFR, EP300, MAPK1, SMAD3, SRC*, and *TP53* overlapped with the genes in CGC. These eight genes were known to participate in cancer progression. The other two genes (*FYN* and *GRB2*) did not match with the CGC database. *GRB2* belongs to druggable genes according to DGIdb. We concluded that *GRB2* had a great ability to participate in the pathogenesis of THCA, and was a possible therapeutic target.

### Enrichment Analysis

**Figure 4:**
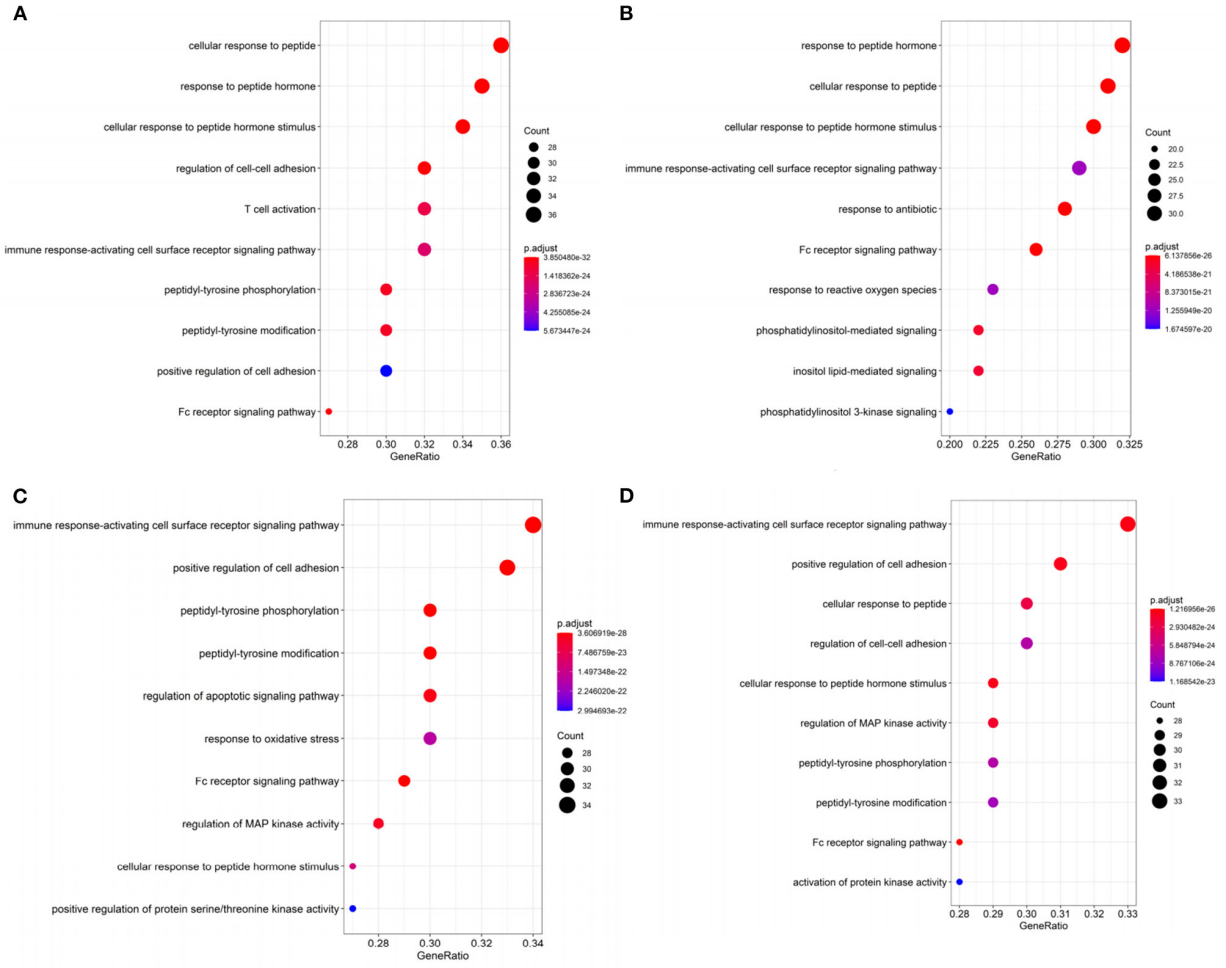
The top 10 gene ontology (GO) terms enrichment of **(A)** BRCA, **(B)** HNSC, **(C)** KIRC, and **(D)** THCA by significant genes with *p* < 0.05 in DriverSubNet.

KEGG and GO enrichment analysis displayed that the top 100 uncovered genes of cancers were significantly enriched in vital KEGG and GO terms, as shown in Supplementary Figure 1, Figure 4, respectively.

In BRCA, the most significantly enriched KEGG term was “Proteoglycans in cancer” (Supplementary Figure 1). Proteoglycans are implicated in regulating cellular growth and differentiation (Filmus et al., 2008). Other enriched terms (e.g., Viral carcinogenesis, ErbB signaling pathway, chronic myeloid leukemia, and prostate cancer) are also related to cancer. The top ranked significantly enriched GO term was peptide associated (Figure 4A). Peptide hormone can negatively regulate iron efflux and is crucial for modulating the growth of breast tumors (Blanchette-Farra et al., 2018). Other enriched terms (e.g., Fc receptor signaling pathway, adhesion) are also related to cancer. Fc receptor can be acted as an indicator for prognosis in many cancers, such as colorectal and lung cancer (Cadena Castaneda et al., 2020). The roles of Fc receptor signaling pathway in BRCA brings forward the need for further studies.

In HNSC, the significantly enriched KEGG term was cancer related, such as proteoglycans in cancer, viral carcinogenesis, and pancreatic cancer. In Figure 4B, “Response to reactive oxygen species” was the enrichment GO term, which can induce oxidative stress (Ma, 2013). Increased reactive oxygen species production involved in multiple cancers through various mechanisms, for example, they can express pro-tumorigenic signaling, and lead to tumor abnormal survival and proliferation, and avail to DNA damage and genetic instability (Moloney and Cotter, 2017). Oxidative stress can contribute to the maintenance of genomic instability during the progression phase of cancer (Hassani et al., 2019) remove. This suggests that oxidative stress has a clinical significance in cancer remove. Moreover, the cellular response to oxidative stress plays crucial roles in cellular adaptation to hypoxic stress remove. Other terms including immune response-activating cell surface receptor signaling pathway, phosphatidylinositol-mediated signaling, Fc receptor signaling pathway, and so on. Moreover, Fc receptor plays a crucial role in NK cell maturation and tumor immunosurveillance (Cadena Castaneda et al., 2020). Immune system play a vital role in HNSC (Mirza et al., 2019). Thus, the top 100 genes in HNSC that we identified were significantly related to cancer.

In KIRC, KEGG pathway annotation indicated that the pathways most enriched in chemokine signaling pathway, neurotrophin signaling pathway, ErbB signaling pathway (Supplementary Figure 1). The top ranked GO term in KIRC was “immune response-activating cell surface (Figure 4C). The top 100 genes identified in KIRC were significantly related to cancer. Other terms including regulation of apoptotic signaling pathway, and Fc receptor signaling pathway, regulation of MAP kinase activity, positive regulation of protein serine/threonine kinase activity were also recorded. Deregulation in apoptotic is a hallmark of cancer (Pistritto et al., 2016). Apoptosis alteration is responsible for tumor development and progression (Pistritto et al., 2016). Other terms, such as response to oxidative stress, cell-cell adhesion, and Fc-gamma receptor signaling pathway, were involved in cancer progression. Through above analysis, these top 100 genes identified in KIRC were related to cancer.

In THCA, KEGG pathway analysis revealed that the top 100 genes were linked with proteoglycans in cancer, chemokine signaling pathway, ErbB signaling pathway, and so on (Supplementary Figure 1). The most significantly enriched GO term was “immune response-activating cell surface receptor signaling pathway” (Figure 4D). This means that the top 100 genes in THCA make a contribution to modulate immune system in cancer. Other enriched terms, such as regulation of cell-cell adhesion and Fc receptor signaling pathway, regulation of MAP kinase activity are associated with cancer progression. Thus, the top 100 genes that we identified were significantly related to cancer.

### Actionable Druggable Genes

**Figure 5:**
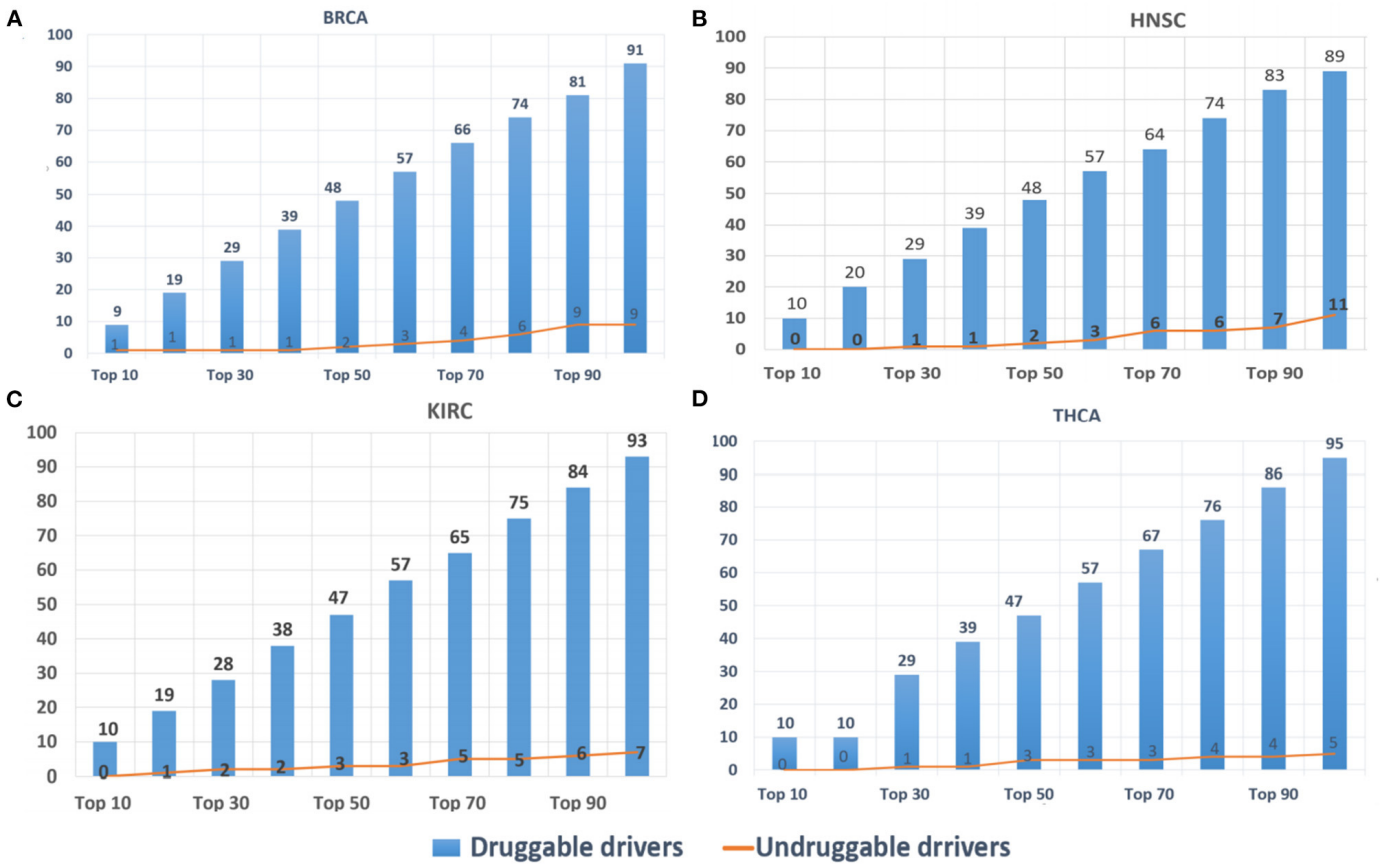
Distribution of top 100 candidate-driver genes from the four cancer gene databases in druggable genes databases. **(A)** BRCA, **(B)** HNSC, **(C)** KIRC, and **(D)** THCA.

DriverSubNet's rankings can guide scientists to decide on drug development and clinical treatment. The top 100 driver genes for BRCA, HNSC, KIRC, and THCA, respectively, were looked-up in DGIdb. Genes with target drug information were considered as druggable driver genes, and the others as undruggable driver genes. The results (Figure 5) indicated that most of the identified driver genes were druggable driver genes. In Figure 5, it was obvious that the proportions of druggable genes increased substantially when the number of genes were increased. Hence, DriverSubNet has the ability of uncovering potential therapeutic targets, tailored to the clinical treatment.

## Discussion

Many methods have been designed to screen driver genes by distinguishing them from passenger genes, but almost all of them have limited sensitivity and specificity. To solve this shortcoming, we constructed the DriverSubNet, which effectively mined the mutation and expression information in PPI network. The algorithm takes into effect of central gene on neighboring DEGs, and mutated frequency. Comparing DriverSubNet with Dawnrank and DriverNet on the four cancer datasets, our results reveal that DriverSubNet achieves better performance than Dawnrank and DriverNet methods in the top 100 gene set. DriverSubNet was able to find well-known genes, such as *EGFR*. In addition, DriverSubNet could also found functional driver genes which have a low mutation rate.

Indeed, to explore the non-CGC candidate genes in the top 100 candidate driver genes by DriverSubNet, we performed literature search, and found that most of non-CGC candidate genes with experimental evidence revealing their relation with cancer. Among the top 10 driver genes identified in BRCA, HNSC, KIRC, and THCA (Supplementary Table 2), overall, seven unique genes (*CDK1, GRB2, YWHAG, SHC1 and PTK2, FYN*, and *TRAF2*) were detected as non-CGC genes. *YWHAG* is critical for maintaining several canonical pathways. miRNAs can directly target *YWHAG*, which has been reported as a tumor suppressor, and participates in the progression in breast cancer, glioblastoma, and lung cancer (Yoo et al., 2016; Wang et al., 2017a,b). *GRB2* encodes protein can activate cell surface receptors in signaling transduction (Giubellino et al., 2008). *GRB2* signaling is associated with cell motility, angiogenesis, and vasculogenesis (Giubellino et al., 2008). These functions make *GRB2* a potential target biomarker to hinder tumor metastasis and local invasion (Giubellino et al., 2008). *SHC1* encoding protein is recruited to tyrosine kinases, which is essential for breast cancer initiation, progression, and metastasis (Ahn et al., 2017). It has implicated that *SHC1* mediate several key signaling pathways in breast cancer (Wright et al., 2019). *PTK2* is a highly phosphorylated kinases in breast cancer (Mertins et al., 2016). Substantial evidence has shown that activated PTK2 expression level links to tumor progression (Fan et al., 2019). In our result, *PTK2* is highly expressed (Fold Change = 1.39) in BRCA samples, which suggests that high PTK2 expression leads to BRCA growth and metastasis. *FYN* is differentially expressed in multiple cancers, and has a correction with cancer progression by controlling cellular motility, cell growth, and death (Elias and Ditzel, 2015). *FYN* is a promising candidate therapeutic marker and may be applied to Fyn-targeted therapy (Elias and Ditzel, 2015). *TRAF2* is reported as an NF-κB-activating oncogene (Shen et al., 2015). *CDK1* can regulate cell cycle progression by executing the G2/M phase transition (Asghar et al., 2015). *CDK1* is the central regulator of cell proliferation and a promising therapeutic target for BRCA (Galindomoreno et al., 2017). Knockout of *CDK1* in mouse experiments revealed that *CDK1* contributed to cellular proliferation (Santamaría et al., 2007). *DLG1* expression associates with the progress of cervical disease (Cavatorta et al., 2017). Through the above analysis, we may find that cancer is heterogeneity that the same driver gene has differential function across cancers, for example, *GRB2* is identified driver gene in four dataset, and *GRB2* expression has a significant survival rate in KIRC, while not in other three cancer types. The findings from this analysis indicate that six genes (Figure 3) which are not in CGC or the independent predictor of poor survival or therapeutic target genes, may contribute to cancer through other mechanisms. Namely, DriverSubNet was able to find these unknown cancer driver genes which could act as potential therapeutic targets and useful prognostic biomarkers for overall survival of patients.

Through performing the KEGG and GO enrichment of these top 100 ranked genes in BRCA, HNSC, KIRC, and THCA, respectively, these drivers were involved in oxidative stress, immune response-regulating cell surface receptor signaling pathway, apoptotic signaling pathway, and immune response-activating cell surface receptor signaling pathway. All of the KEGG and GO terms play important roles in the response to cancer.

Although the present study shows various positive results, it has certain limitations as well. Future validation using multiple cancer types is warranted. In addition, the present study did not attempt to use the synonymous mutations (Wen et al., 2016) and indels (insertions and deletions) (Yue et al., 2019), which have been found to regulate tumorigenesis via various mechanisms (Yue et al., 2019; Zhang and Xia, 2020). We will attempt to integrate these somatic mutation data in our future work.

In conclusion, we have designed an effective and no parameter algorithm, termed DriverSubNet, for prioritizing cancer driver genes by integrating somatic mutational, expression, and PPI network. As indicated by the evaluation of four cancer datasets, DriverSubNet consistently outperformed Dawnrank and DriverNet methods in terms of precision, recall, and F1 score. Further, it was able to identify potential driver genes that have not been documented, but might be important driver genes. Thus, DriverSubNet acted as a useful tool for the identification of driver genes by subnetwork enrichment analysis. However, studies with larger multiple cancer types and by including synonymous mutations and indels will be helpful in further development of this method.

## Data Availability Statement

The original contributions presented in the study are included in the article/Supplementary Material, further inquiries can be directed to the corresponding author/s.

## Author Contributions

DZ conceived the algorithm, designed the method, carried out the experiments, analyzed the data, and drafted the manuscript. DZ and YB refined the idea, polished the English expression and revised the paper, and participated in the design and revision of the research. All authors read and approved the final manuscript.

## Conflict of Interest

The authors declare that the research was conducted in the absence of any commercial or financial relationships that could be construed as a potential conflict of interest.

## Supplementary Material

Supplementary Figure 1 The top 10 gene ontology (KEGG) terms enrichment of **(A)** BRCA, **(B)** HNSC, **(C)** KIRC, and **(D)** THCA by significant genes with *p* < 0.05 in DriverSubNet.

Supplementary Table 1 The details of the dataset.

Supplementary Table 2 A list of top 100 candidate-driver genes of four datasets.

## Abbreviations

TCGA: The Cancer Genome Atlas
SCNAs: Somatic Copy Number Alterations
THCA: Thyroid Carcinoma
KIRC: Clear Cell Kidney Carcinoma
BRCA: Breast Cancer
HNSC: Head-Neck Squamous Carcinoma
CGC: Cancer Gene Census
FG: Functional Set
DEGs: Differentially Expressed Genes
PPI: Protein-Protein Interaction.
