# Supplementary figures and images for: DriverSubNet: A Novel Algorithm for Identifying Cancer Driver Genes by Subnetwork Enrichment Analysis
Source: Front Genet. 2021 Feb 19;11:607798. doi: 10.3389/fgene.2020.607798 (PMC7933651; doi:10.3389/fgene.2020.607798)

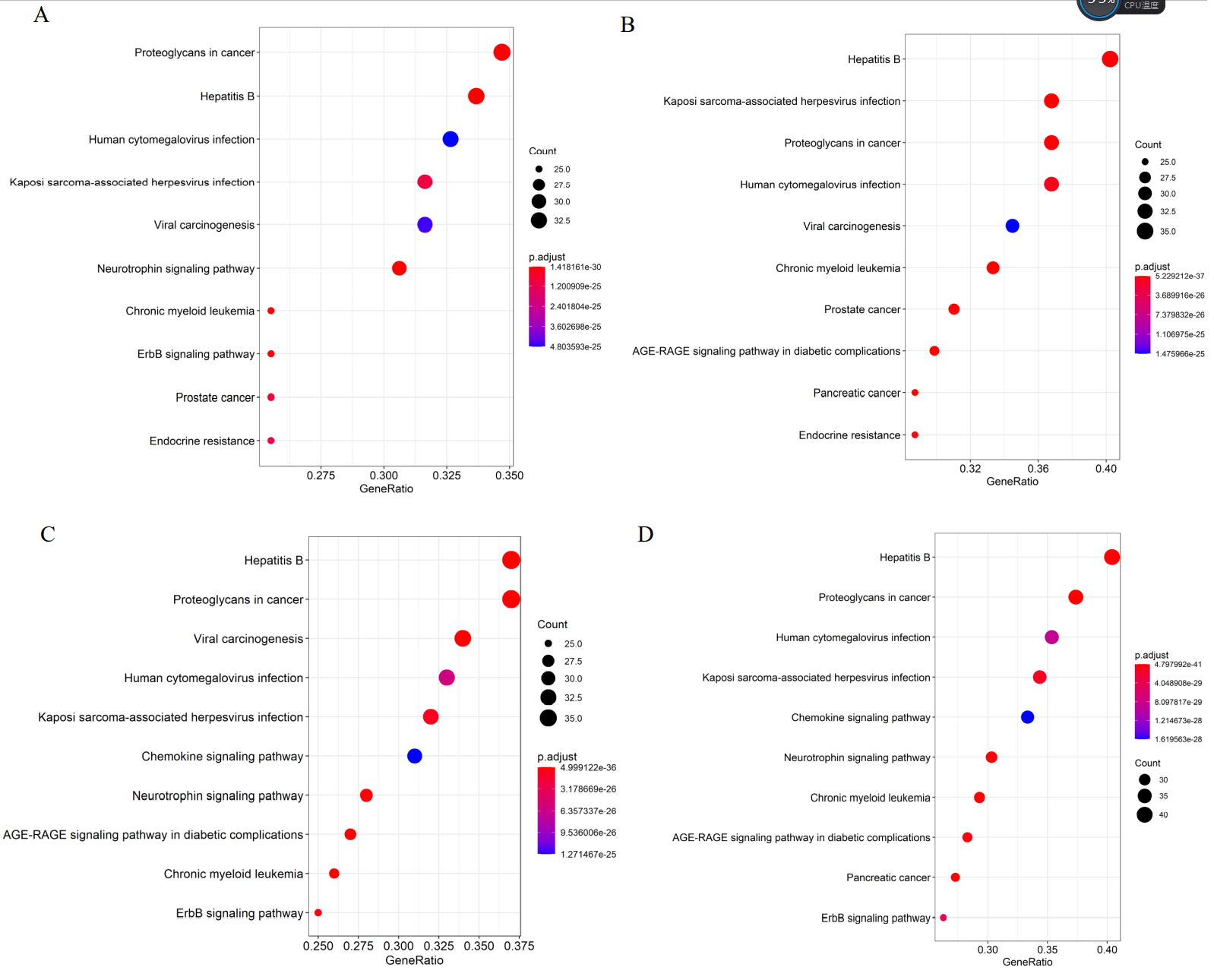

Supplement: Supplementary Figure 1 — The top 10 gene ontology (KEGG) terms enrichment of (A) BRCA, (B) HNSC, (C) KIRC, and (D) THCA by significant genes with p < 0.05 in DriverSubNet. [file Image_1.TIF]
