# Supplementary material for: DriverSubNet: A Novel Algorithm for Identifying Cancer Driver Genes by Subnetwork Enrichment Analysis
Source: Front Genet. 2021 Feb 19;11:607798. doi: 10.3389/fgene.2020.607798 (PMC7933651; doi:10.3389/fgene.2020.607798)
Supplement: Supplementary Table 1 — The details of the dataset. [file Table_1.DOCX]

Table S1. The details of the data

| Tumor type | Number of tumor expression samples (The number of genes) | Number of normal expression samples (The number of genes) | Number of somatic mutation samples (The number of genes) | Samples of tumor expression∩somatic samples |
| --- | --- | --- | --- | --- |
| THCA | 513（20530） | 59（20530） | 515 （24861） | 513 |
| HNSC | 522（20530） | 44（20530） | 530 （25027） | 522 |
| KIRC | 534（20530） | 72（20530） | 529 （24870） | 526 |
| BRCA | 1097（20530） | 114（20530） | 1099 （25327） | 1092 |
